# Resolution in Two-Photon Imaging: A Local Manifestation of Entanglement

**DOI:** 10.1021/acsphotonics.5c01310

**Published:** 2025-09-27

**Authors:** T. Gregory, E. Toninelli, P.-A. Moreau, S. P. Mekhail, O. Wolley, K. Roberts, J. Bělín, S. M. Barnett, M. J. Padgett

**Affiliations:** † School of Physics and Astronomy, 3526 University of Glasgow, Glasgow, G12 8QQ, United Kingdom; ‡ Cambridge Consultants, 29 Cambridge Science Park, Milton Road, Cambridge, CB4 0DW, United Kingdom; § Department of Physics, National Cheng Kung University, Tainan 70101, Taiwan; ∥ Center for Quantum Frontiers of Research and Technology, National Cheng Kung University, Tainan 70101, Taiwan; ⊥ Central European Institute of Technology, Brno University of Technology, Purkyňova 656/123, 612 00 Brno, Czech Republic; # Faculty of Mechanical Engineering, Institute of Physical Engineering, Brno University of Technology, Technická 2896/2, 616 69 Brno, Czech Republic

**Keywords:** Quantum Enhanced Imaging, Resolution Enhanced Imaging, Entanglement, SPDC Photon-Pairs, Single-Photon Sensitive Detection, EMCCD Camera

## Abstract

The resolution of a classical imaging system is limited by diffraction. This limit can be overcome, for example, by implementing various forms of localization microscopy in which the center of a fluorescence distribution is estimated to an accuracy scaling with the square root of the number of detected photons, $\sqrt{N}$ . In quantum imaging the object can be illuminated using correlated photon-pairs, leading early work to suggest that a $\sqrt{2}$ improvement could be obtained as a result of averaging the position of *N* = 2 events. However, similar to quantum lithography, which relies upon quantum illumination using entangled photon-pairs and two-photon absorption, the minimum resolvable feature size is reduced by a factor of 2, not just $\sqrt{2}$ . Quantum imaging schemes can also lead to a factor of 2 improvement. By using a similar source of correlated photon-pairs to illuminate an object, a single-photon sensitive camera to detect the photon-pairs, and an image processing algorithm to record and sum the bisector positions of the transmitted photon-pairs, we realize a similar factor of ×2 improvement in image resolution, surpassing that of most earlier quantum imaging work.

## Graphical abstract

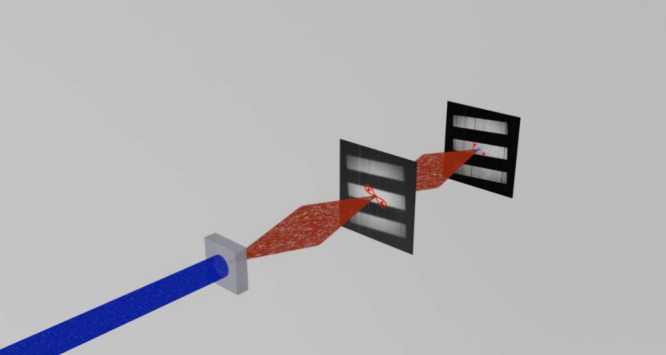

## Introduction

A perpetual challenge in optical physics is to overcome the diffraction limited spatial resolution of an imaging system which is set by the wavelength of the imaging light and the numerical aperture of the lens system. An example of resolution enhanced imaging is single-molecule localization microscopy (SMLM) where the position of isolated fluorophore molecules are localized by measuring the center of the detected fluorophore emission.^1^ The accuracy with which the center of the fluorescence can be localized increases with the number of photons, *N*, measured, allowing the diffraction limit to be beaten by a factor of $\sqrt{N}$ . This $\sqrt{N}$ improvement is referred to as the standard-quantum-limit (SQL).

In quantum optics experiments parametric down conversion produces photon-pairs (*N* = 2), labeled signal and idler, which are correlated in their position and anticorrelated in their momenta. In quantum ghost imaging the idler photons interact with the object and the spatially correlated signal photons are detected on a spatially resolved detector.^2^ In quantum illumination^3^ the photons that comprise the pair can be used to perform imaging either in the image plane, in which both photons interact with the object, or the far-field plane, in which one of the photons interacts with the object, of the downconversion source. The photon-pairs are then detected using a single-photon sensitive spatially resolving detector array, thereby revealing the position or momentum correlations. It has been shown, moreover, that suitable quantum illumination can improve the limits in the resolution of beam displacements by a factor of *N*.^4^ A related, but nonimaging, example is the proposal for quantum lithography in which the interference of entangled photon-pairs is used to obtain a factor 2 improvement in resolution of lithographic features by using a photoresist sensitive to 2-photon absorption.^5,6^ The improvement by a factor of *N* is referred to as the Heisenberg limit for a quantum-correlated N-photon system.^7,8^ Heisenberg scaling of the uncertainty in phase estimation can be achieved using N00N states^9^ and improvements beyond the standard quantum limit have also been demonstrated in the context of phase microscopy using these states.^10^ Using a combination of quantum imaging using correlations between photon-pairs and classical image scanning microscopy, an improvement of a factor of 2 in resolution has been realized over the purely classical technique for a system capable of performing microscopy of cell samples.^11^

By performing N-photon detection of a N-photon state for SPDC photon-pairs (*N* = 2) in a quantum imaging system, the resolution can be defined as that of a coherent system with an effective illumination wavelength of λ_*s*,*i*_/2. However, many quantum enhanced resolution imaging experiments do not demonstrate that entangled states are required to achieve the observed resolution enhancement and as such there may be classical configurations that could achieve an equivalent enhancement.^12^

Within the quantum domain, it has been demonstrated that the spatial correlations between photon-pairs produced by spontaneous parametric downconversion (SPDC) can be used to surpass classical limits.^13–20^ For example, optical centroid estimation of detected photon-pairs generated by SPDC was proposed to improve the resolution of an imaging system.^21–23^ Illuminating an object using a pump beam and performing centroid estimation on the detected SPDC photon-pairs generated by this pump enabled the recovery of a resolution equivalent to that of using the pump beam directly, i.e., a factor of 2 improvement in the resulting image resolution.^24^ Such a technique has an advantage should the detector not be sensitive to the wavelength of the pump beam used to illuminate the sample. Previously, we have also utilized correlated photon-pairs generated using SPDC within a nonlinear crystal cut for type-I downconversion to probe an object placed in the image plane of the crystal. We performed centroid estimation of the position of the detected photon-pairs and noted a resolution improvement compared to the direct imaging case.^25^

When applying any algorithm, for example, performing an AND-operation^26,27^ or identifying two-photon events,^28^ to select correlated events, there will always be additional uncorrelated event pairs that are also selected. Uncorrelated event pairs can arise from combinations of detector noise events and the accidental overlap of neighboring photon events that appear to be correlated and so are also selected by the algorithm. Adding these accidental correlations degrades the enhancement from the theoretically achievable maximum.

An example of this reduction in enhancement as a result of accidental correlations is shown in our previous work demonstrating background light removal and our ability to distinguish the true object, a bird, illuminated using a SPDC source from the spoof object, a cage, illuminated using a thermal source.^27^ In that work we perform an AND-operation to select spatially correlated photon-pair events detected in anticorrelated pixel positions on an detector array. In performing the AND-operation, accidental AND-events are also selected by the algorithm and degraded the ability to distinguish the true object from the spoof object. We have since demonstrated an algorithm for the removal of image component created by these accidental correlations and achieved an improvement of up to 11× in the ability to distinguish the true object from the spoof object.^29,30^ Similarly, in our work on resolution enhancement using photon-pairs,^25^ the algorithm used to select photon-pairs and calculate the bisector also collects accidental pair-events. These accidental pair-events are between uncorrelated detector events, whether they be photons or detector noise, and prevent the full quantum resolution enhancement by using spatially entangled photon-pairs from being realized. Our ambition here is that by removing the contribution to the image from the uncorrelated event-pairs we might achieve the full theoretical quantum resolution advantage. In order to remove these uncorrelated event-pairs, we subtract the centroid estimated image constructed using light that is uncorrelated *C*_bis_ from the centroid estimated image constructed using light that is quantum correlated *Q*_bis_. We show that once the image arising from accidental correlations is subtracted, we obtain an image with a resolution enhancement approaching a factor of 2 in the midrange frequencies of the calculated Spatial Frequency Response (SFR) curve assessed at SFR50.

There exist other quantum enhanced imaging schemes that have advantages in the context of resolution and these results are discussed here. Harnessing entangled states of light in the form of SPDC beams that are first-order incoherent but exhibit second-order coherence, an advantage in resulting image resolution can be realized. Using a ptychographic imaging scheme could enable the acquisition of high-resolution intensity and phase images with enhancement of up to a factor of *N* with appropriate treatment such as that presented in Aidukas et al. (2019),^31^ although a resolution advantage was not quantified in that work. Also, using the properties of SPDC incoherence, quantum holography techniques have been demonstrated to present an advantage in resolution when compared to classical holography using coherent light and a factor of 1.84 has recently been reported by Defienne et al.^32^ We also note the use of spatially entangled photon-pairs by Defienne et al. (2022)^33^ to sample at a doubled sampling frequency resulting in an image resolution increase and also enables the removal of aliasing effects in addition to the capacity for rejecting background noise. This technique serves to double the effective pixel resolution, whereas in our work we present an optical resolution improvement.

In coincident detection schemes utilizing SPDC photon-pairs, an improvement in resolution has also been realized relative to coherent laser light illumination at the signal and idler wavelength^34,35^ and also relative to using the incoherent SPDC signal beam.^36^ In these works, it is described that while there is an improvement in image resolution, the expected enhancement may not be fully realized. This is because the transmission function for the SPDC coincidence case is a convolution of the magnitude of the transmission functions of the lens system for the signal and idler photons of wavelength λ_*s*,*i*_, which is not equal to the transmission function for a pump photon of wavelength λ_*p*_ = λ_*s*,*i*_/2. Similar schemes have also been used to perform phase imaging using incoherent SPDC illumination.^37^ In a more recent implementation of a coincident detection scheme microscopy was performed by scanning an object under the illumination of a focused multimode SPDC beam. Comparing the coincidence image to the singles image a factor of $∼\sqrt{2}$ improvement in image resolution was reported^38^ and by using a camera to perform multiplexed detection, as opposed to scanning a similar factor of ∼2 enhancement was also reported.^39^ However, in their imaging scheme the signal and idler photons from a SPDC photon-pair are split in the far-field into two beams with one acting as the reference and the other probing the object, so only one photon in the photon-pair is probing the object.

In this work, we attribute the realized factor of 2 enhancement to the fact that the photon-pairs are entangled and therefore are not only correlated in their position, but also anticorrelated in their momentum. Hence, we argue that in terms of resolution, exceeding the standard quantum limit and approaching the Heisenberg limit is a manifestation of quantum entanglement between the photon-pairs. The relationship between momenta of the photons has some parallels with the relationship between illumination scattered light in Fourier ptychography^31,40^ or twin illumination beams in structured light microscopy.^41^ Recent work that also utilizes the position-momentum spatial correlations between entangled photon-pairs and imaging one in the far-field plane and the other in the image plane of the sample has enabled phase microscopy with phase-resolution comparable to that of classical schemes while also rejecting background light.^42^

For the images we present here in this work, the object is in the image plane of the downconversion source and so the quantum and classical data is obtained by illuminating the target with the signal and the idler beams. These beams are first-order incoherent; however, the SPDC photon-pairs exhibit second-order coherence by virtue of their correlations.^43^ As such, the comparison is between classical incoherent imaging and our bisector image with accidentals subtracted. This subtraction method can be used to reject noise and reveal the correlations between SPDC photon-pairs whether that be spatial correlations for quantum-enhanced imaging or temporal correlations for Hong-Ou-Mandel experiments. However, this method cannot be used to measure a violation of a Bell inequality which requires measuring the product of detected intensities for unique pairs of photons.^44^

## Materials and Methods

### Experimental Design

The data reanalyzed in this work is that presented in ref 25 and obtained using the experimental setup shown in Figure 1. A 355 nm laser was used to pump a 3 mm thick β-barium borate (BBO) nonlinear crystal cut for type-I degenerate downconversion to generate degenerate SPDC photon-pairs at 710 nm. The pump beam was attenuated to ∼3 mW/mm^2^ in order to optimize the detected SPDC illumination level. After passing through the nonlinear crystal, the residual pump beam was removed using a pair of dichroic mirrors which reflected the pump beam out of the system while transmitting the downconverted light. The pump beam, which is in the UV, must be removed in order to prevent it from generating fluorescence in the subsequent optical components which would only serve to add uncorrelated light to the camera images. The plane of the crystal was imaged onto an object using a pair of lenses $f_{L_{1}}=150\mathrm{mm},\mathrm{and},f_{L_{2}}=100\mathrm{mm}$ , and this object plane was then reimaged onto the detector plane using an aperture-limited optical 4f system which comprises lenses $f_{L_{3}}=100\mathrm{mm},\mathrm{and},f_{L_{4}}=100\mathrm{mm}$ . The resolution of this second lens system was defined by the placement of an aperture in the far-field of the object. The detector array used was an Andor 888 EMCCD camera of 13 μm × 13 μm pixel size and 100% fill-factor. An interference filter centered on the degenerate wavelength of 710 nm with a bandwidth of 10 nm and a top-hat profile was placed on the front of the detector array such that only photons near the degenerate wavelength were transmitted to the detector.

**Figure 1:**
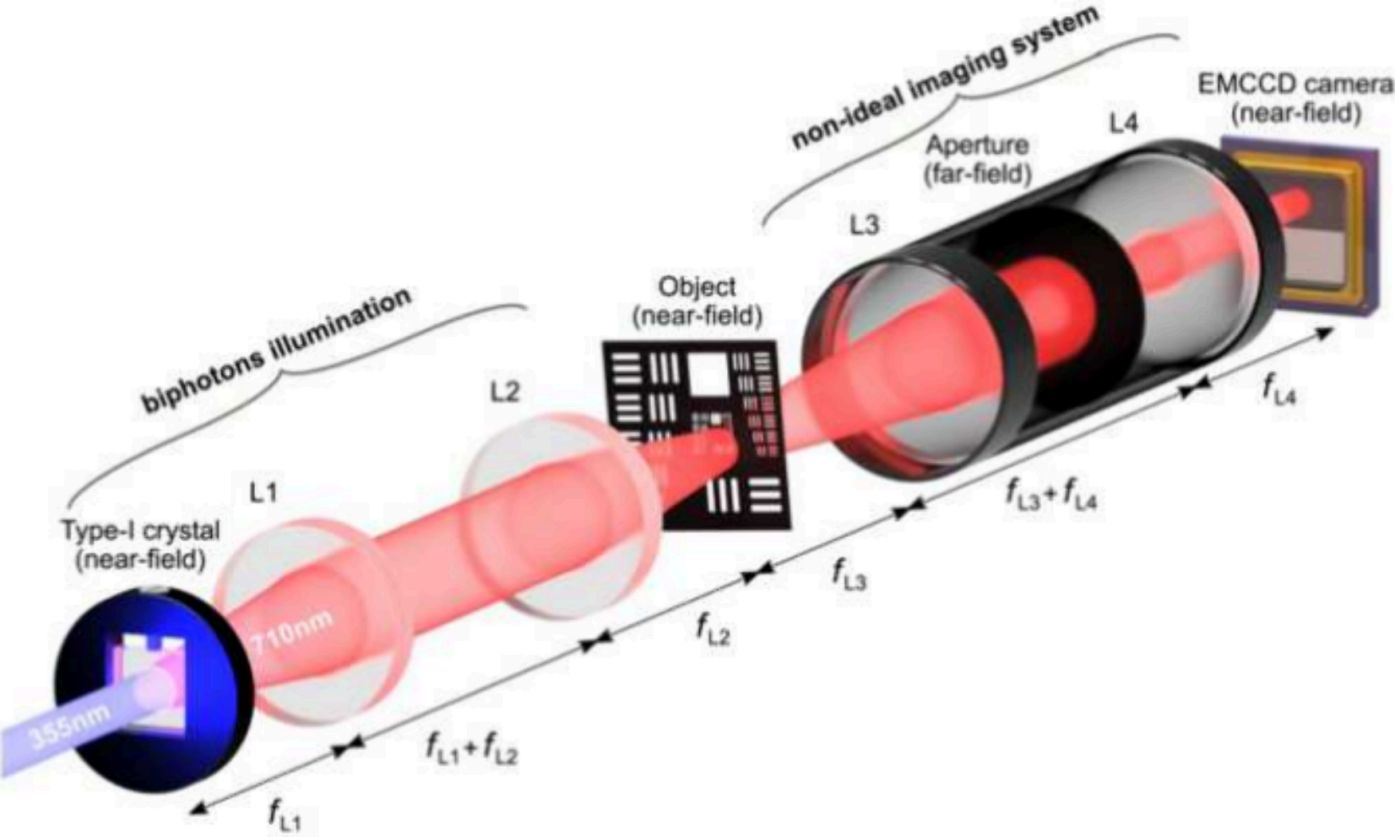
Schematic of the resolution enhancement experimental setup. The optics of our resolution-enhanced imaging scheme consists of a source of spatially correlated photons (labeled as “photon-pair illumination”), an object, a nonideal imaging system (in our case, an NA-limited system), and a single-photon sensitive EMCCD camera. The image plane of the crystal and of the object are imaged onto the plane of the detector. An aperture placed in the far field of both the crystal and the object is used to tune the diffraction limit of the nonideal imaging system. Figure reprinted with permission from ref 25. Copyright 2019 Optical Society of America.

By placing an ND filter (optical density of ND = 2) after the downconversion crystal, the correlations within the downconverted beam were largely lost and therefore the attenuated downconversion beam can be approximated as an incoherent beam that does not exhibit spatial correlations. The pump beam power was accordingly modified such that the frames comprising the correlated photon-pairs data upon which the quantum centroid was calculated, and the uncorrelated photon data upon which the classical centroid was calculated, contained the same mean number of events. The event rate was set such that there were on average 100 photon events and 700 camera noise events per frame (356 × 356 pixel^2^), resulting in a total event rate of <0.001 events per pixel per frame for the frames used to calculate both the quantum centroid and classical centroid images.

The expected strength of the spatial correlations generated using our downconversion source can be calculated using eq 1 in which α = 0.455.^45–49^ In the image plane of the downconversion crystal, the strength of the position correlation is then 12.42 μm which corresponds to a standard deviation of σ = 0.96 pixels, and a FWHM ≈ 2.25 pixels for pixel dimension of 13 μm × 13 μm.

1 $$\sigma_{x_{1}-x_{2}}=\sqrt{\frac{\alpha L\lambda_{p}}{\pi }}$$

### Analysis

To obtain an image by centroid estimation of photon-pairs, the same analysis is performed as in Toninelli et al. (2019).^25^ In that work each EMCCD frame was binarized using a threshold on the analogue counts in order to select photon events. A series of 11 different 3 × 3 pixel kernels were then applied to each frame to select unique event-pairs as shown in Figure 2 reproduced from Toninelli et al. (2019).^25^ The centroids of these selected detector event-pairs were then used to build a estimation of the quantum enhanced image. This is performed both for light that is uncorrelated *C*_bis_ and for light that is correlated *Q*_bis_. As described in the preceding section, the uncorrelated beam is generated by placing a neutral density filter (optical density of ND = 2) after the downconversion crystal, the losses introduced significantly reduce the strength of the spatial correlations. In both the correlated and uncorrelated cases, the mean flux of downconverted photon events at the detector is equalized by adjusting the pump power.

**Figure 2:**
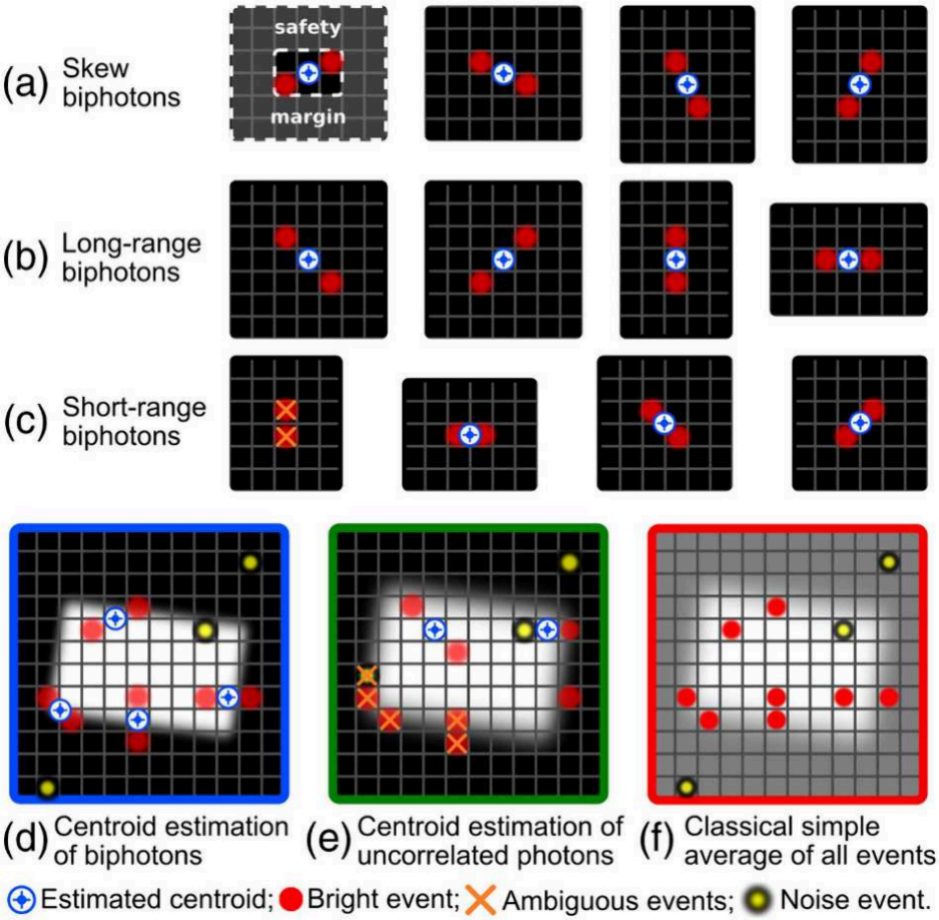
Series of 3 × 3 pixel kernels used to identify unique detector pair-events and calculate centroids. For each kernel, a safety margin of two pixels around the kernel is enforced to avoid calculating centroids of ambiguous pair-events. One of the kernels is not used, as it corresponds to the direction of detector readout and so is associated with charge smearing. Figure reprinted with permission from ref 25. Copyright 2019 Optical Society of America.

In this present work, we subtract the uncorrelated from the correlated image *Q*_bis_ – *C*_bis_ to obtain the centroid difference or subtracted image. To remove negative values and prevent modifying the frequency response, the minimum value is added back to the difference image prior to further operations including conversion to PNG files upon which the SFR is measured.

In an EMCCD camera operating with EM-gain, a single photon event undergoes amplification by a stochastic process. A threshold is set, above which a photon-event is defined to have occurred. This operation thereby linearizes the data by setting a single photon-event to a single image event-count. This is valid for a low pixel occupancy rate ≪ 1 photon events per pixel per frame which comprises the illumination regime used here. It is these binarised frames with a linear response that are used to construct the images presented in this manuscript. The binarisation operation also does not filter for certain spatial frequencies. The subtraction operation serves to remove the image luminance that corresponds to uncorrelated event pairs and so the centroid difference image represents the luminance that originates only from the truly spatially correlated photon-pairs. The subtraction operation also does not filter for certain spatial frequencies, as both high spatial-frequency information (edge regions) and low spatial-frequency information (smooth regions) are preserved in the centroid difference or subtraction image.

## Theory

We argue that the observed factor of 2 enhancement in the image resolution observed in our work presented here and others is a consequence of the photon-pairs produced in the down-conversion process being entangled. This entanglement means that in the image plane of the down-conversion crystal the signal and idler photons are strongly correlated in their transverse position and also that they are anticorrelated in their transverse momentum. These properties mean that the photon-pairs so produced can be used to demonstrate the famous Einstein-Podolsky-Rosen (EPR) paradox^50^ associated with the failure of local realism (or nonlocality for short).^51^ Nonlocality is not the only physical consequence of entanglement, and we shall see that the factor of 2 enhancement in image resolution is such a (local) feature of the entanglement.

The simplest argument for enhanced imaging, and the possibility of the observed factor of 2 enhancement in image resolution, lies in the fact that the entangled state of each photon-pair depends on the properties of the shorter wavelength photon that created them. This, together, with the fact that the resolution limit is inversely proportional to the wavelength indicates the possibility of the factor of 2 enhancement, as this is what would be observed using photons of half the wavelength from the pump. It is now well-known, moreover, that such photon-pairs can be used to achieve a factor of 2 resolution enhancement in photolithography if the medium exposed is processed by two-photon absorption.^5,52,53^ In these processes, the two photons combined behave, essentially, as a single pump photon would, and in particular, they can be made to acquire a collective two-photon phase shift as if they had a single de Broglie wavelength.^54,55^ What is unexpected in our work, and requires explanation, is how the factor of 2 enhancement appears without requiring two-photon absorption. We address this question here. Our explanation for the factor of 2 enhancement derives either from the image-plane transverse position correlation or from the transverse momentum anticorrelation. The combination of these is, of course, a feature of the entanglement of the photons. We find that we can derive the resolution enhancement using either the position entanglement or the transverse momentum entanglement. We investigate the former in some detail and supplement this with a shorter argument in terms of transverse momentum entanglement.

### Transverse Position Entanglement

To make our argument it suffices to consider just a single transverse dimension and to represent the object by a mask-function, *M*(*x*), representing the transmission amplitude of the object. In this, we follow the example of Abouraddy et al.^37^ and Saleh et al.^43^ Henceforth, references to calculated bandwidth or range of transverse spatial frequencies are defined as the standard deviation of the spatial frequencies transmitted by the aperture. To set a reference resolution, we determine the effective spatial bandwidth that can be obtained using single (plane-wave) photons with the same frequency as those produced in our down-conversion process. The limiting resolution is determined by the range of transverse spatial frequencies in the Fourier transform of *M*(*x*) that can pass through an aperture placed in the Fourier plane of the object. Let the limits of this aperture correspond to the spatial frequencies −κ/2 and κ/2 of the form presented in Figure 3a. It then follows that the greatest range of spatial frequencies is simply associated with a flat amplitude across the aperture, for which the width of the spatial bandwidth distribution, B­(*k*_(1 photon)_), is presented in eq 2. It is this restricted range of transverse wave-vectors that determines the achievable resolution.

$$B\left(k_{\left(1\,\;\mathrm{photon}\right)}\right)={\left(\frac{\int_{-\kappa /2}^{\kappa /2}dk\,\;k^{2}}{\int_{-\kappa /2}^{\kappa /2}dk}\right)}^{1/2}=\frac{\kappa }{\sqrt{12}}$$ 2

**Figure 3:**
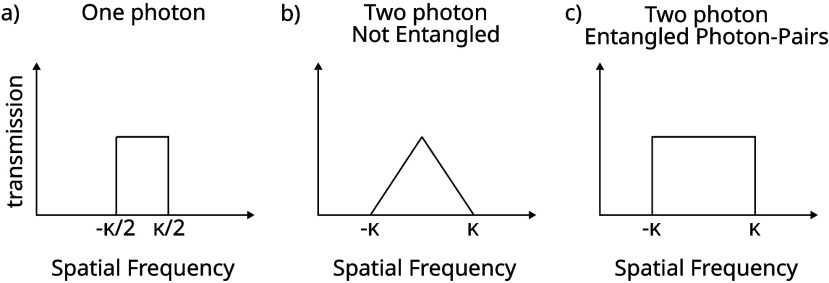
Visualization of spatial frequencies transmitted by the aperture placed in the Fourier plane of the source for different illumination types.

It is instructive to compare this with the limiting resolution that can be achieved with two (uncorrelated) photons prepared in our plane wave. Our imaging method selects the mean position of the two detected photons and this depends on the sum of the two wave-vectors for the individual photons transmitted in the Fourier plane. This sum takes values between −κ and κ; however, a flat distribution of transverse wave-vectors for each of the two uncorrelated single photons corresponds to a triangular distribution for their sum with its peak at the origin and falling to zero at −κ and κ of the form presented in Figure 3b. It follows that the width of the possible values for the sum of the two wave-vectors and therefore the width of the spatial bandwidth distribution, B­(*k*_(2 photon)_), increases corresponding to a factor of $\sqrt{2}$ in comparison with the single-photon result as given in eq 3.

$$B\left(k_{\left(2\,\;\mathrm{photon}\right)}\right)=\frac{\kappa }{\sqrt{6}}=\sqrt{2}B\left(k_{\left(1\,\;\mathrm{photon}\right)}\right)$$ 3

Alternatively, we can understand this, simply, as the effective width arrived at for a pair of independent wave-vectors. The situation when using the bisector of two photons is more subtle and requires us to consider the state of the two photons and the effect of this on the filtering that occurs in the Fourier plane. Consider first the effect of the mask on our single plane-wave photon. The mask modifies the state of the photon to the state in eq 4.

$$\psi_{\left(1\,\;\mathrm{photon}\right)}\left(x\right)=M\left(x\right)$$ 4

This state then propagates to the Fourier plane where it has the form M̃(k) , which is the Fourier transform of *M*(*x*). For two plane-wave photons the state in the object plane which becomes as per eq 5. In the Fourier plane, this becomes the state $\mathrm{M̃}\left(k_{1}\right)\mathrm{M̃}\left(k_{2}\right)$ . These states representing the two uncorrelated single photons can be factorized and therefore is not an entangled state.

$$\psi_{\left(2\,\;\mathrm{photon}\right)}\left(x_{1},x_{2}\right)=M\left(x_{1}\right)M\left(x_{2}\right)$$ 5

For photon-pairs from our SPDC source, the positions of two photons in the object plane (image plane) coincide, so we can write the two-photon state in the form presented in eq 6 where Δ­(*x*_1_ – *x*_2_) is a function with a single sharp peak at *x*_1_ = *x*_2_.

6 $$\psi_{\mathrm{SPDC}}\left(x_{1},x_{2}\right)=M\left(\frac{x_{1}+x_{2}}{2}\right)\Delta \left(x_{1}-x_{2}\right)$$

The form of this function is clearly entangled in the positions of the two photons as it does not factorize into the product of a function of *x*_1_ and a function of *x*_2_. As a result, the wave function in the Fourier plane takes the approximate form $\mathrm{M̃}\left(k_{1},k_{2}\right)\neq \mathrm{M̃}\left(k_{1}\right)\mathrm{M̃}\left(k_{2}\right)$ . The value of *k*_1_ + *k*_2_ varies between −κ and κ, and if we again spread the values evenly over this range, then as per eq 7 we find a doubling of the spatial frequency bandwidth for a single-photon plane wave of the form presented in Figure 3 c).

$$B\left(k_{\mathrm{SPDC}}\right)=\frac{\kappa }{\sqrt{3}}=2B\left(k_{\left(1\,\;\mathrm{photon}\right)}\right)$$ 7

It is the values of *k*_1_ + *k*_2_ that determine the average position of the photons detected in the image plane and so our method of image reconstruction benefits from this potential doubling of the bandwidth with the associated enhancement in resolution. It is essential to realize that this enhancement is necessarily a consequence of the position entanglement inherent in the entangled state:^51^ the enhanced spread in transverse wave-vectors is a consequence of the (near) colocation of the two photons in the object (image) plane, as expressed in the function Δ­(*x*_1_ – *x*_2_), while the imaging information is contained in the function $M\left(\frac{x_{1}+x_{2}}{2}\right)$ . In Figure 3, a depiction of the spatial frequencies that are transmitted by the aperture for one-photon, two-photon non entangled, and two-photon entangled light is presented. This theoretical depiction may not exactly match the form of the experimental results due to physical constraints including finite pixel size, aperture centering, and image shot-noise.

### Transverse Momentum Entanglement

It is in the very essence of entanglement that strong correlations in one observable are accompanied by strong correlations (or anticorrelations) in an incompatible one. Indeed nonlocality paradoxes and tests are largely based on this property.^56,57^ It is reasonable to ask, therefore, if the above discussion of the enhanced resolution, based on transverse position entanglement, has a corresponding explanation in terms of the well-known transverse momentum anticorrelation associated with down-converted photon-pairs. We show here that such an explanation does indeed exist. We start by recalling that the phase-matching condition is met for spontaneous parametric downconversion when one of the photons has transverse momentum *q*, and its partner has transverse momentum −*q*, i.e., the SPDC photon-pairs exhibit momentum anticorrelations.^58^ Two such photons will produce, in the Fourier plane, the combined two-photon wave function $\int_{-\infty }^{\infty }dq\mathrm{M̃}\left(k_{1}+q\right)\mathrm{M̃}\left(k_{2}-q\right)$ , which is clearly entangled in the transverse momentum and accesses a wider range of spatial frequencies than are found for two photons in the same plane wave. This situation is reminiscent of structured illumination microscopy^41^ and, indeed, of related techniques associated with Fourier ptychography.^40,59^ In structured illumination microscopy, however, a number of distinct illuminations are required to construct the enhanced image. In our experiment it is the entanglement that provides a superposition of different *q* values and hence the required information for the enhanced resolution is present is each photon-pair. As with the transverse position, the required combination of the momentum anticorrelations and the spread of transverse momenta is a clear consequence of the entanglement between the photons and we would not achieve the resolution without both features and hence without momentum entanglement.

In the derivation that we have presented here, our aim was to make the explanation as simple as possible, and for this a single dimensional treatment suffices. Moreover, it is because our simple analysis uses a single transverse spatial dimension that the standard deviation suffices to illustrate the observed effect. Our aim here is not to provide a detailed theoretical/computational model of the observed effects, but rather a readily accessible explanation of the underlying physical mechanism (entanglement) that is responsible for the observed enhancement of the resolution. We have checked our conclusions in two transverse dimensions and found that they hold, but have opted (both for brevity and clarity) to present our simpler analysis based on a single dimension. Readers are also invited to read^24^ for a 2D theoretical derivation that finds a similar factor of 2 enhancement in resolution.

## Results

The SFR curves derived in this present work to quantify the resolution enhancement were calculated in the same manner as previously^25^ by imaging a clear optical-path USAF resolution target orientated at an angle of 5° relative to the readout direction of the detector array, and a slanted edge test. The spatial frequency response of the centroid estimation of photon-pair images constructed from uncorrelated illumination *C*_bis_, correlated illumination *Q*_bis_, and their difference *Q*_bis_ – *C*_bis_ are calculated using SFR3 by P. Burns,^60–64^ which is based on ISO 12233:2017.^65^

Images of the USAF resolution target calculated by summing the centroid positions of pair-events over ten million frames are presented in Figure 4 and the associated SFR curves are displayed in Figure 5. The red curve corresponds to the classical average image, the green curve corresponds to the classical centroid image for which uncorrelated illumination is used, the blue curve corresponds to the quantum centroid image for which correlated illumination is used, the pink curve corresponds to the centroid difference-image *Q*_bis_ – *C*_bis_. Error bars represent standard error on the mean calculated from ten images each constructed using one million frames. The purple curve represents the total average SFR curve for the centroid difference-image calculated from the sum of the analysis from all ten million frames. The improvement in resolution is calculated at SFR50, SFR90, and SFR10. The SFRA is calculated as the area under the SFR curve up to the cutoff frequency corresponding to SFR10 (SFR = 0.1).^66^

**Figure 4:**
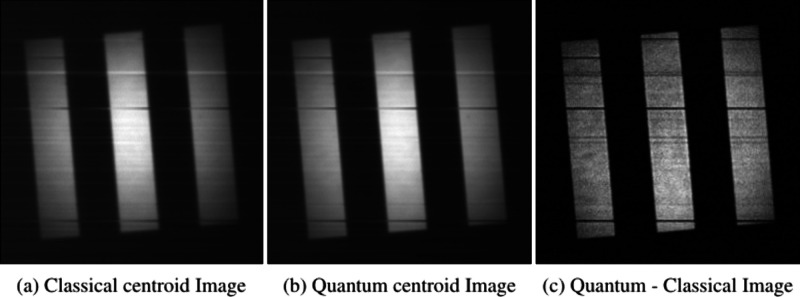
Slanted edge test target images constructed from the full set of ten million frames. (a) Classical centroid image constructed from centroid events using uncorrelated light *C*_bis_, (b) quantum centroid image constructed from centroid events using correlated light *Q*_bis_, and (c) quantum-classical image obtained by subtracting the classical centroid image from the quantum centroid image *Q*_bis_ – *C*_bis_.

**Figure 5:**
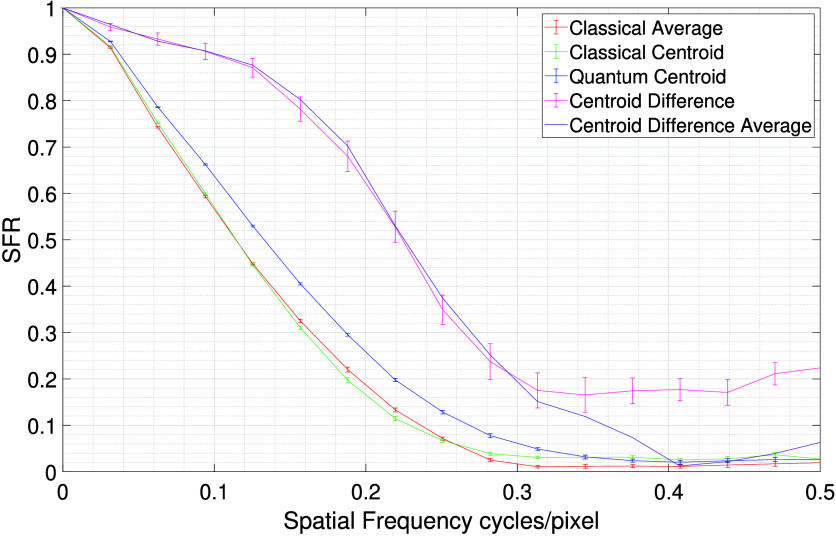
SFR curve for slanted edge object. The SFR curve for images of the slanted edge object calculated from 10 million frames shows an improvement in the SFR for the centroid difference image (pink line) over both the quantum centroid image (blue line) and the classical centroid image (green line). The classical average (red line) is also displayed for comparison. Error bars are calculated on ten blocks of one million frames. The purple line represents the SFR curve for the total centroid difference-image calculated using the full ten million frames.

The calculated values of these various metrics upon which resolution is assessed are presented in Table 1 in which it can be seen that the centroid difference-image is enhanced relative to both the originally reported quantum centroid image, and also the classical centroid image. The classical centroid image is equivalent in resolution to the direct classical image. Between the classical centroid and the total centroid difference-image, the advantage in SFR50 is a factor of 1.97, SFR10 a factor of 1.56, SFR90 a factor of 2.93, and SFRA a factor of 1.93. For the work presented here the classical image is obtained by illuminating the target with the SPDC signal and idler beams. These beams are first order incoherent; however, the SPDC photon-pairs exhibit second order coherence by virtue of their correlations. As such, the comparison is between classical incoherent imaging and our bisector image with accidentals subtracted. The transfer function of the system is different when comparing the incoherent to the coherent illumination case the incoherent transfer function being the convolution of the coherent transfer function with itself, and therefore with increasing coherence this decreases the cutoff frequency but improves the frequency response for spatial frequencies below this value. This is consistent with the increase in the SFR50 by a factor of 2, while the SFR10 cutoff frequency only increases by $\sqrt{2}$ .

**Table 1:** Image Resolution Parameters from the SFR Graphs Presented in Figure 5 ^a^

	SFR50	SFR10	SFR90	SFRA
Classical Average	0.1142 ± 0.0005	0.2364 ± 0.0018	0.0339 ± 0.0005	0.0978
Classical Centroid	0.1144 ± 0.0017	0.2291 ± 0.0031	0.0346 ± 0.0017	0.0966
Quantum Centroid	0.1329 ± 0.0016	0.2685 ± 0.0030	0.0375 ± 0.0006	0.1121
Centroid Difference	0.2243 ± 0.0291	NaN	0.0996 ± 0.0150	NaN
Centroid Difference (Total)	0.2255	0.3579	0.1014	0.1865

a The SFR50, SFR10, and SFR90 values are calculated by interpolating between the two points on either side of SFR = 0.5, 0.1, and 0.9, respectively. SFRA values are calculated by integrating the area between the SFR curves and the line corresponding to the SFR10 on the corresponding averaged SFR curve calculated using the full 10 million frames. Values for SFR50, SFR10, and SFR90 presented in rows 1–4 in this table are calculated using 10 blocks of one million frames to compute the average SFR curve and standard error on the mean which are then used to calculate the interpolation values and uncertainties presented in this table.

In Figure 5 it can be seen that SFR curve is improved for the centroid difference-image (pink line) when compared to both the quantum centroid image (blue line) and the classical centroid image (green line). Both the classical centroid and the quantum centroid SFR curves are smooth as each image contains many events; however, for the centroid difference-image the reduced number of events in the image results in increased noise in the images and an increased noise floor for the SFR curve. For the centroid difference-image, the SFR curve meets the noise floor at ∼0.3 cycles/pixel for the average SFR for ten images constructed using one million frames. The solid purple line represents the response of the total centroid difference-image calculated using the full ten million frames. This increase in data serves to reduce the shot noise in the images on which the SFR curve is calculated and thereby suppresses the increased noise floor, which is evident at higher spatial frequencies for the pink curve. The cut off frequency for centroid difference image is ∼0.4 cycles/pixel.

Our improvement in the spatial frequency response by performing the subtraction operation is because the average classical centroid image can be removed leaving only the pair-events that correspond to spatially correlated SPDC photon-pairs. The accidental centroid events in both the classical centroid and quantum centroid image are a consequence of random correlations, which is related to the number of events per pixel per frame. The accidental centroid pair-events comprise detector noise, environmental noise, and single SPDC photons for which the pair-photon was subject to losses. For an equal photon flux, and a low quantum efficiency, the accidental centroid event rate is equivalent for both the correlated and the uncorrelated illumination. The random nature of these accidental correlations would otherwise lead to a blurring of the image. Subtracting the classical centroid from the quantum centroid serves to remove the random correlations that contribute to the blurring of the quantum centroid image and enables the quantum-enhanced resolution advantage to be realized. The classical centroid and quantum centroid images are constructed from the same number of frames with the same event rate and therefore the accidental centroid rates are equivalent, which enables the subtraction to be performed.

The SFR measure using the slanted edge method uses the entire edge present in the region of interest to calculate a supersampled Edge Spread Function (ESF). This is then used to calculate the SFR by taking the derivative to obtain the Line-Spread Function (LSF) and smoothing prior to performing a Fourier transform, for which a detailed explanation can be found in ref 64. As such, the measure is not of the resolution at a single point on the edge but is calculated by sampling along its length. As can be seen in Figure 6 the resolution does vary spatially across the three bars of the USAF test target. For the central portion of the image and the central four edges the SFR response of the system is similar. The average SFR50 enhancement for the centroid difference image compared to the classical centroid image is a factor of 1.89× for this central region. For the outermost two edges (red and orange hue ROIs and lines in Figure 6) there is degradation in the SFR response of the system for the acquired images; however, the enhancement between the classical centroid and centroid difference images is still present and for the case of the leftmost bar the enhancement is up to a factor of 2.40×. A factor of 2 still falls within the uncertainty in the ratio calculated from the interpolated SFR50 values for the extreme ratios of 2.40× and 1.71×. Overall this means that the average value of SFR50 enhancement across the six regions of interest is a factor of 1.98× which is in agreement with the above results. A factor of 1.94× is found when disregarding the two outliers. SFR50 values and the ratio for each of the ROIs is presented in Table 2. The outer edges present reduced resolution because these regions are furthest from the center of the spherical singlet lenses used so are subject to increased aberrations which degrade the image resolution. Also, the illumination is not uniform across the bars and so there are fewer centroid events in these regions which serves to increase the shot-noise which affects the SFR measurement and curves at higher spatial frequencies. The region of interest used to calculate the plots in Figure 5 is the purple/magenta region highlighted in Figure 6b,c. This region was chosen because this portion of the image has a continuous edge that is not interrupted by high contrast camera readout lines which can affect the SFR measurement.

**Figure 6:**
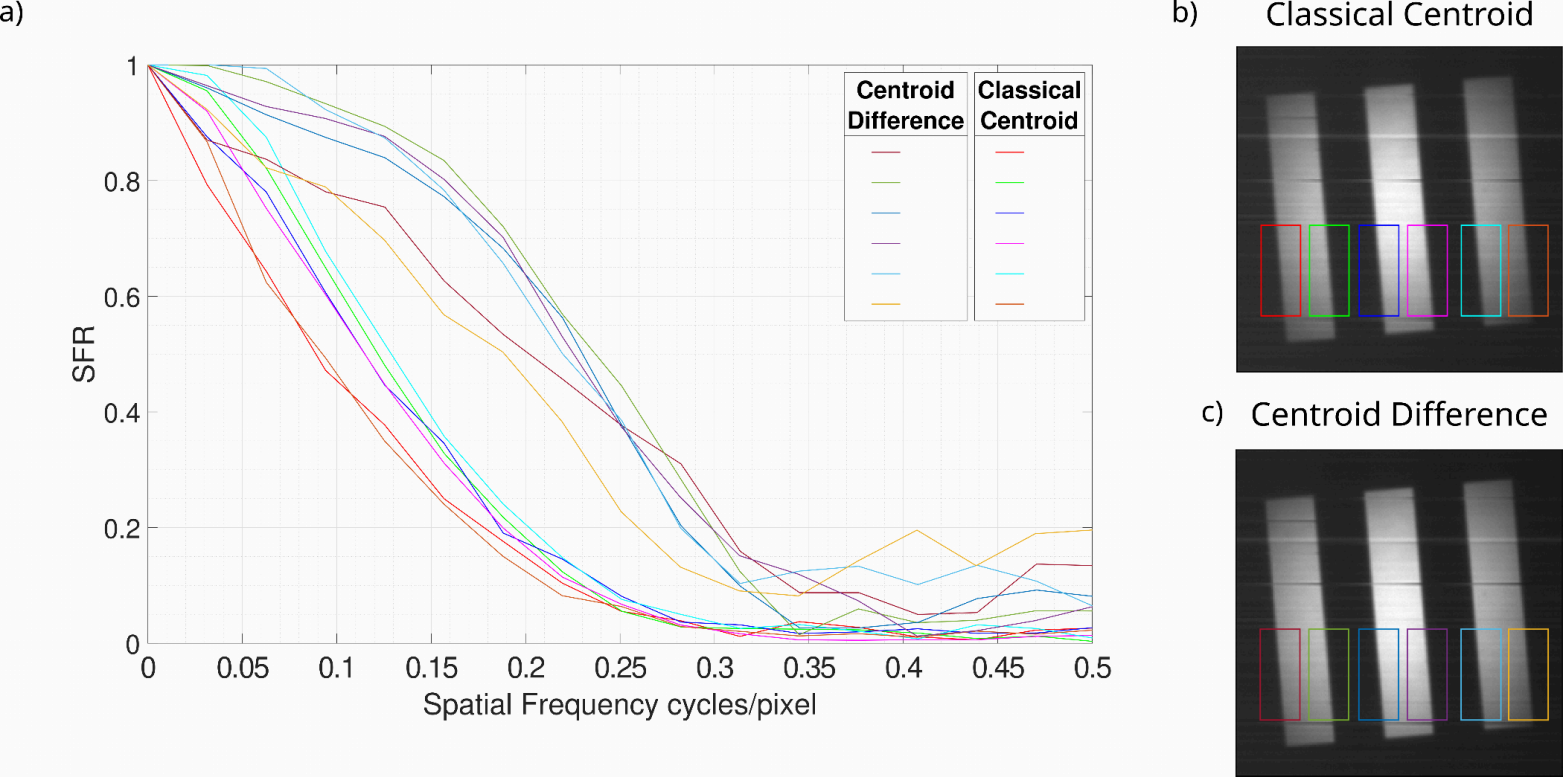
Spatial variance in the resolution assessed using SFR curves for regions of the slanted edge object. SFR curves calculated for the classical centroid image and the centroid difference image for different comparable regions of the image to investigate spatial variance in the resolution. These curves are calculated using images constructed from ten million frames.

**Table 2:** Table Presents the SFR50 Values for the Calculated from the SFR Curves for the Range of ROIs Presented in Figure 6 ^a^

	ROI 1	ROI 2	ROI 3	ROI 4	ROI 5	ROI 6
Classical Centroid	0.0883	0.1213	0.1171	0.1144	0.1293	0.0926
	±0.0114	±0.0107	±0.0173	±0.0128	±0.0323	±0.0152
Centroid Difference	0.2124	0.2267	0.2351	0.2242	0.2215	0.1795
	±0.0299	±0.0330	±0.0284	±0.0291	±0.0135	±0.0192
Ratio	2.4041	1.8689	2.0074	1.9601	1.7127	1.9385
	±0.4603	±0.3182	±0.3829	±0.3356	±0.4409	±0.3800

a The SFR50 values are calculated by interpolating between the two points on either side of SFR = 0.5 for each of the SFR curves. Values in this table are calculated using ten blocks of one million frames to compute the average SFR curve and standard error on the mean which are then used in the interpolation for the calculation of the SFR50 value and interpolation uncertainties presented in this table.

For both the bars and real-world objects, the centroid difference images contain fewer events than the quantum centroid image and the classical centroid images. However, in addition to an increased image resolution and contrast, the SNR for the central bar in the centroid difference image is in fact increased relative to both the classical and quantum centroid images. The ratio $\sqrt{N}/\sigma$ improves as the uncorrelated noise, and importantly the fluctuations thereof, are reduced in the classical and quantum bisector images, and then removed in the centroid difference image. However, the shot-noise limit is not reached for any of the presented data sets. Image metrics for the bars images are presented in Table 3. It is well-known that quantum light can also be harnessed to improve upon image noise by rejecting uncorrelated light and sensor noise. The shot-noise on the transmissive portions of the image can always be further improved upon simply by acquiring more data, while the image resolution cannot directly be improved by this method. There would be an indirect improvement in the high spatial frequencies by smoothing the image as demonstrated by the differences between the pink and purple SFR curves representing the centroid difference and centroid difference average images shown in Figure 5. Any visible noise in our images is simply circumstantial and could have been visually cleared by using longer acquisitions or acquiring more photon-pair events.

**Table 3:** Table Presents the Mean, Standard Deviation, SNR for the Central Bar and Right Adjacent Dark Region of the Bars Objects Presented in Figure 4

	Bright Mean	Bright σ	SNR	$\sqrt{N}/\sigma$	Dark Mean	Dark σ	Dark SNR
Classical Image	123134.12	17086.95	7.21	0.02	49067.16	3147.60	15.59
Classical Centroid	10118.73	2049.38	4.94	0.05	2186.80	215.66	10.14
Quantum Centroid	11725.72	2188.88	5.36	0.05	2127.25	210.67	10.10
Centroid Difference	2077.99	348.56	5.96	0.13	411.45	69.78	5.90

Further to reporting an improvement in the SFR curve derived from a slanted edge object, we present the improved results for a number of real-world objects in Figures 7, 8, and 9 that were originally presented in our previous work.^25^ In these images the resolution enhancement provided by performing the centroid operation and subsequent subtraction of the accidental classical centroids can be seen especially when observing the edge of the wasp wing and the lamp filament objects in Figures 7 and 8. For the glass fibers object shown in Figure 9 some fibers moved between the separate quantum and classical data acquisitions thereby creating some voids in the difference image after subtraction. Imaging of real-world objects demonstrates the potential of this method to perform resolution enhanced imaging in real-world scenarios.

**Figure 7:**
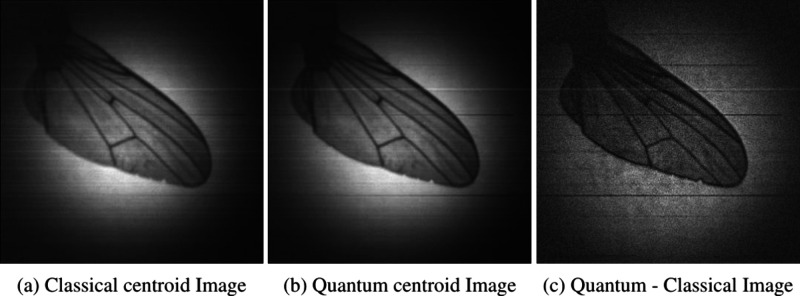
Classical, quantum, and quantum minus classical centroid images of a wasp wing.

**Figure 8:**
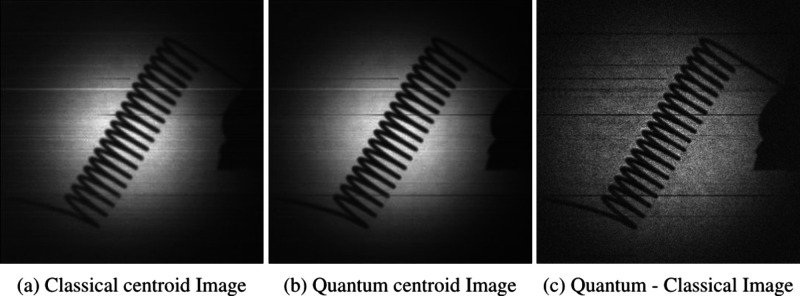
Classical, quantum, and quantum minus classical centroid images of a lamp filament.

**Figure 9:**
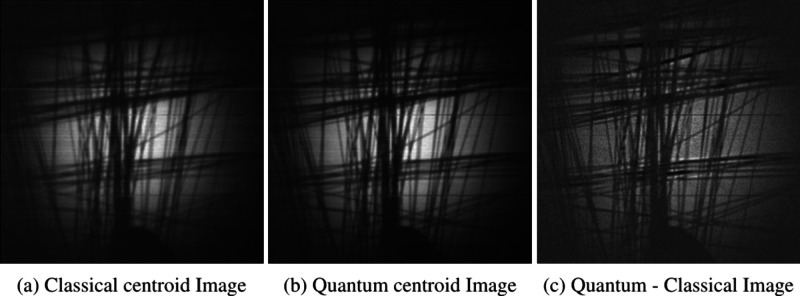
Classical, quantum, and quantum minus classical centroid images of glass fibers.

## Discussion

We report an imaging system using centroid estimation of spatially correlated photon-pairs (*N* = 2) produced by spontaneous parametric downconversion. By adopting an algorithm that calculates the image as the difference between the centroid image from correlated pairs and a centroid image from accidental pairs, we realize a factor of 2 improvement in the spatial frequency for which the image contrast has dropped by 50%. Our insight in this work is not only to derive an algorithm to obtain this resolution enhancement but to realize that this improvement is a manifestation of the position-momentum entanglement of the SPDC photon-pairs.

The robustness of our algorithm is confirmed by applying our imaging system and algorithm to image a number of real-world objects where qualitative resolution enhancements can also be observed. Such imaging techniques make quantum illumination schemes more viable as the technology is developed for real-world applications because uncorrelated noise sources that reduce image contrast, distinguishability, and resolution can be removed.
